# Diversity, Chemical Constituents and Biological Activities of Endophytic Fungi Isolated from *Schinus terebinthifolius* Raddi

**DOI:** 10.3390/microorganisms8060859

**Published:** 2020-06-07

**Authors:** Paola dos Santos da Rocha, Vanessa Marina Branco Paula, Silvia Cristina Figueira Olinto, Edson Lucas dos Santos, Kely de Picoli Souza, Leticia Miranda Estevinho

**Affiliations:** 1 Research Group on Biotechnology and Bioprospecting Applied to Metabolism, Federal University of Grande Dourados, Rodovia Dourados Itahum, Km 12, 79804-970 Dourados, MS, Brazil; paolarocha.biologa@gmail.com (P.d.S.d.R.); silviafigueira@ufgd.edu.br (S.C.F.O.); edsonsantosphd@gmail.com (E.L.d.S.); 2 Polytechnic Institute of Bragança, Agricultural College of Bragança, Campus Santa Apolónia, 5301-855 Bragança, Portugal; vanessapaula@ipb.pt (V.M.B.P.); leticia@ipb.pt (L.M.E.)

**Keywords:** pink pepper, endophytes, phenolic compounds, volatile compounds, antioxidant, antibacterial

## Abstract

*Schinus terebinthifolius* Raddi is a medicinal plant widely used for the treatment of various diseases. The secondary metabolites responsible for the pharmacological properties can be produced directly by the plant or by endophytic fungi. The objective of this study was to evaluate the diversity of endophytic fungi of different parts of *S. terebinthifolius* and to identify chemical compounds produced by endophytes and their antioxidant and antibacterial activities. For this, fruits, stem bark and roots were dried, ground and placed in fungal growth medium. The selected endophytes were grown and subjected to extraction with ethyl acetate. DPPH, FRAP, β-carotene bleaching and antimicrobial assays were performed. The phylogenetic tree was elaborated, encompassing 15 different species. The fungal extracts showed hydroxybenzoic acids and 1-dodecanol as predominant compounds. All fungal extracts exhibited antioxidant activity. The fungal extracts exhibited bactericidal and bacteriostatic activities against Gram-positive and Gram-negative bacterial ATCC strains and against methicillin-resistant nosocomial bacteria. Among the 10 endophytic fungi evaluated, the extract of the fungus *Ochrocladosporium elatum* showed higher phenolic content and exhibited higher antioxidant and antibacterial activities in all tests. Together, the results increase the known diversity of *S. terebinthifolius* endophytic fungi, secondary metabolites produced and their antioxidant and antibacterial activities.

## 1. Introduction

*Schinus terebinthifolius* Raddi (Anacardiaceae), commonly called Brazilian pepper tree and known in Brazil as the pink pepper, is a medicinal plant native to Brazil. The ethnopharmacological use of this species includes the treatment of urinary and respiratory infections, skin sores and ulcers, tumors, diarrhea and arthritis [1,2]. In addition, the fruits of this plant are widely used as a spice in human food.

Bioprospecting of new natural products is a resource that has been widely used in science for medicinal purposes [3,4,5]. Endophytic fungi are organisms that colonize plant tissues and, in symbiosis, use the primary metabolites produced by plants for the production of secondary metabolites, which may exert protective, maintenance and growth functions in the host plant [6]. These products can be isolated and applied for various purposes. Among the active compounds isolated from endophytic fungi is penicillin, an antibiotic produced by the species *Penicillium notatum* [7], taxol and vincristine, recognized antineoplastic agents produced by the endophytic fungi *Taxomyces andreanae* and *Fusarium oxysporum* [8]. Thus, plant endophytic fungi are sources of bioactive substances and are, therefore, targets for the detection of new compounds with pharmacological activities of interest, such as antioxidant and antibiotic activities [9,10].

Currently, oxidative stress, a result of excess non-neutralized reactive species in the body, has been indicated as one of the main deleterious factors in several types of diseases, such as diabetes, obesity, cardiometabolic diseases, neurodegenerative diseases and even some types of cancer [11,12]. Endophytic fungi produce several substances that act as antioxidants, among which are phenolic compounds [13].

In addition, there is growing concern worldwide regarding the control of bacterial infections, including those caused by *Klebisiella pneumoniae*, *Salmonella enteriditis* and *Staphylococcus aureus*. Gram-negative bacteria *K. pneumoniae* and *S. enteritidis* are linked to a wide variety of infections. *K. pneumoniae* is mainly associated with nosocomial infections, urinary tract infections, pneumonia and blood infections and causes pyogenic liver abscesses, endophthalmitis and meningitis [14]. *S. enteritidis* is primarily responsible for food-borne contamination, causing infections in the gastrointestinal tract, and the development of systemic diseases [15]. *S. aureus*, a Gram-positive bacterium, is a human pathogen that has mechanisms to acquire resistance to multiple antimicrobial agents and is constantly a source of human infections, including infective endocarditis, skin infections, osteomyelitis, lung infections, gastroenteritis and urinary tract infections [16,17,18].

The antioxidant [19,20,21,22,23] and antimicrobial [21,23,24,25,26] activities of different parts of *S. terebinthifolius* have been described using different pharmacological models. However, only the endophytic fungi of *S. terebinthifolius* leaves [24,27,28] have been identified. In this context, this study aimed to identify the diversity of endophytic fungi present in fruits, stem bark and root of *S. terebinthifolius* as well as the chemical compounds and the antioxidant and antibacterial activities of the extracts of these endophytes.

## 2. Materials and Methods

### 2.1. Endophytic Fungi Isolation

Endophytic fungi were isolated from fruits, stem bark and root of *S. terebinthifolius*. To this end, 1 g of pulverized material was sterilized in 5 mL of 70% ethanol for 2 min, 5 mL of 4% sodium hypochlorite for 5 min and washed two times with 5 mL of sterile distilled water. At each step, the material was centrifuged at 3000 rpm for 2 min and the supernatant discarded. The pulverized and sterilized material was inoculated into Petri plate containing solid medium, malt extract agar (MEA) with 10% tartaric acid or potato dextrose agar (PDA) with 10% tartaric acid or rose bengal medium. Plates were incubated at 25 °C for 7 days. Colonies of the fungi were later isolated in new plates. The endophytic fungi were isolated for identification.

### 2.2. Extraction, Amplification and DNA Sequencing

The molecular identification of endophytic fungi was carried out by the laboratory ^®^STABVIDA, Lda, Caparica, Portugal. To extract DNA from endophytic fungi, one colony from each culture was suspended in 10 μL of sterile Phosphate Buffered Saline (PBS 1x, pH 7.4). DNA extraction from the cell culture was stored in FTATM Micro Card Indicator. Amplification of a segment containing the ITS and D1/D2 regions was performed by PCR. Subsequently, we proceeded to the sequencing of the amplified product (DNA Sanger sense and antisense). Sequencing was performed using ITS (ITS1/ITS4) and D1/D2 (NL1/NL4) specific primers. Species were identified from each BLASTed consensus sequence against NCBI nucleotide databases. Phylogenetic tree was constructed with the aid of the program CLC Sequence Viewer 7.8.1. The identified endophytic fungi were cooled in MEA 5% glycerol medium, with identification of ESA-FE 1 to 15 (Escola Superior Agrária of Bragança-Fungos Endofíticos).

### 2.3. Endophytic Fungi Extract

The extract of the endophytic fungi was performed on a basis reported by Kjer et al. [29]. The 10 endophytic fungi that presented growth superior to 5% were selected and inoculated in extract of malt broth for 2 weeks. The pre-inoculum was prepared in Erlenmeyer flasks containing 300 mL of MEA by adding three agar-grown mycelial disks grown on agar (6 mm) of each culture isolated. To obtain the fungal extracts, all samples were macerated with 250 mL of ethyl acetate at 25 °C for 24 h and then filtered and fractionated in separatory funnel with 200 mL of ethyl acetate and 100 mL of water distilled. The ethyl acetate extracts of the endophytic fungi were concentrated using a rotary evaporator under vacuum under reduced pressure at 40 °C. The crude concentrates were further stored at −20 °C.

### 2.4. Phenolic Compounds

The phenolic compounds were determined according to the method of Pinela et al. [30], with microplate modifications. The fungal extract at 2 mg/mL (10 µL) was mixed with 0.1% HCl (10 µL, prepared in 95% ethanol) and 2% HCl (180 µL). After 15 min, the absorbance was measured at 280, 320 and 360 nm. The absorbance (A) at 280 nm was used to estimate the total phenolic content (hydroxybenzoic acids), while A320 nm was used to determine the tartaric esters (hydroxycinnamic acids) and A360 nm was used to determine the flavonoids (flavonols). Gallic acid was used to calculate the standard curve (50–500 µg/mL), and the results were expressed as mg of gallic acid equivalents (GAE)/g of extract. Caffeic acid was used to calculate the standard curve (50–500 µg/mL), and the results were expressed as mg of caffeic acid equivalents (CAE)/g of extract. Quercetin was used to calculate the standard curve (30–300 µg/mL), and the results were expressed as mg of quercetin equivalents (QE)/g of extract. The assays were carried out in triplicate.

### 2.5. Antioxidant Activity

#### 2.5.1. DPPH Assay

The 2,2-diphenyl-1-picrylhydrazyl radical (DPPH) scavenging activity was evaluated according to the method described by Bobo-García et al. [31]. For this experiment, 20 μL of the fungal extract (2 mg/mL) was mixed with 180 μL of 150 µM DPPH solution in 80% ethanol. The mixture was incubated at room temperature in the dark for 40 min. The absorbance was measured at 515 nm. Trolox was used to calculate the standard curve (100–1000 µg/mL), and the results were expressed as mg of trolox equivalents/g of extract. As a control, 20 μL of the solvent (80% ethanol) was incubated with 180 μL of the DPPH solution. Three independent experiments were performed in triplicate. The percent inhibition was calculated from the control using the following equation:

DPPHscavenging activity (%) = (1 − Abs_sample_/Abs_control_) × 100, (1)

For fungal extracts with inhibition higher than 50%, the inhibitory concentration values of 50% (IC_50_) were calculated. Ascorbic acid and hydroxybutylanisole (BHA) were used as standard antioxidants. The final concentrations of the extract and standard antioxidants ranged from 1 to 100 μg/mL.

#### 2.5.2. FRAP Assay

The ferric reducing antioxidant power (FRAP) assay was evaluated according to the method described by Ustundag et al. [32]. For this experiment, 20 µL of fungal extract (2 mg/mL) was mixed with 280 µL of FRAP reagent. The FRAP reagent contained 1 mL of 10 mmol/L 2,4,6-tri(2-pyridyl)-s-triazine (TPTZ) solution in 40 mmol/L HCl, 1 mL of 20 mmol/L iron(3+) trichloride hexahydrate and 10 mL of 0.3 mol/L acetate buffer (pH = 3.6). The mixture was incubated at room temperature in the dark for 30 min. The absorbance was measured at 593 nm. Ammonium sulphate and iron (II) (30–300 µg/mL) was used to calculate the standard curve (100–1000 µg/mL), and the results were expressed as mg of Fe II equivalents/g of extract. Three independent experiments were performed in triplicate. For fungal extracts with activity higher, the half maximal effective concentration (EC_50_) value was determined, for which different concentrations of the extract (200–2000 µg/mL) were used. Ascorbic acid and hydroxy-butylanisole (BHA) were used as reference antioxidants (20–200 µg/mL).

#### 2.5.3. β-carotene Bleaching Assay

β-carotene bleaching inhibition was evaluated according to the method described by Koleva et al. [33]. For this experiment, 1 mg of β-carotene was dissolved in 5 mL of chloroform. β-carotene chloroform solution (1 mL) was added with a pipette to a boiling flask that contained 20 mg of linoleic acid and 200 mg of Tween 40. Chloroform was removed using a rotary evaporator at 40 °C, and 50 mL of oxygenated distilled water was slowly added to the flask with vigorous agitation to form an emulsion. For this assay, 250 µL of the emulsion was added to 30 µL of the fungal extract (2 mg/mL) in microplate. The absorbance measurements made at 492 nm immediately after the addition of the emulsion to the extract as well as again after 120 min. The microplate was placed in an agitating at 50 °C. As a control, 30 μL of the solvent (80% ethanol) was incubated with 250 μL of the emulsion. Three independent experiments were performed in triplicate. The percent inhibition was calculated from the control using the following equation:

β-carotene bleaching inhibition (%) = (1 − Abs_sample_/Abs_control_) × 100, (2)

for fungal extracts with inhibition higher than 50%, the IC_50_ were calculated, for which different concentrations of the extract (200–2000 µg/mL) were used. Ascorbic acid and hydroxybutylanisole (BHA) were used as reference antioxidants (20–200 µg/mL).

### 2.6. Antibacterial Activity

Antibacterial capacity was assessed according to the microplate method described by Molla et al. [34], against methicillin-resistant strains. Two strains of Gram-negative bacteria were used (*Salmonella enteritidis* ESA 111 and *Klebsiella pneumoniae* ESA 232) and a strain of Gram-positive bacteria (*Staphylococcus aureus* ESA 206). As reference, strains of the American Type Culture Collection (*Staphylococcus aureus* ATCC 43300, *Klebsiella pneumoniae* ATCC 13883 and *Salmonella enteritidis* ATCC 13076). Initially, the bacterial strains were inoculated on a sterile nutrient agar plate and incubated at 37 °C for 24 h. Subsequently, prior to the assay, bacterial strains were subcultured into “overnight” nutrient broth.

Fungal extracts (0.1–1 mg/mL) were diluted using the serial microdilution method in nutrient broth. To determine the minimum inhibitory concentration (MIC), 30 μL of 0.08% rezasurin was used. Then, 20 μL of bacterium, MacFarland scale (absorbance 0.3–0.4 at 540 nm) was inoculated. Two independent experiments were carried out in triplicate. Methanol 0.5% was used as a negative control. Gentamicin was used as a reference (0.0001–1 mg/mL). All plates were incubated at 37 °C for 24 h.

To evaluate the minimum bactericidal concentration (MBC), 20 μL of bacterial growth negative wells were seeded by plating on nutrient agar containing plates incubated at 37 °C for 24 h. The result with growth below 10 colonies was considered bactericidal and, more than 10 colonies were considered bacteriostatic.

### 2.7. Volatile Compounds (GC-MS)

Analysis of volatile compounds was performed using a Varian 3800 gas chromatograph equipped with a Varian Saturn 2000 mass spectrometer and an electron impact ion source (EI). The constituents were separated on a Sapiens-Wax MS capillary column (30 m, 0.15 mm i.d., film thickness 0.15 μm). The temperature program was initially set to 60 °C held for 2 min, gradually increased to 234 °C at a rate of 3 °C/min and finally to 260 °C at a rate of 5 °C/min. Helium 49 gas (Praxair) was used as carrier gas at a constant flow of 1.3 mL/min. The injection port, transfer line and temperatures of the ion source were adjusted to 250 °C. Seventy-seven electron volts of ionization energy were used, and the mass scan interval was defined from 3 to 260 m/z in full sweep with an interval of 610 ms. The injection was performed in splitless, maintained for 30 s. The solvent delay time was set at 3 min. The data was processed in the MS Data Review program (v.6.9.3). All volatile components were identified by comparing retention indices and mass spectra obtained from pure standards.

## 3. Results

### 3.1. Identification of Endophytic Fungi

**Figure 1:**
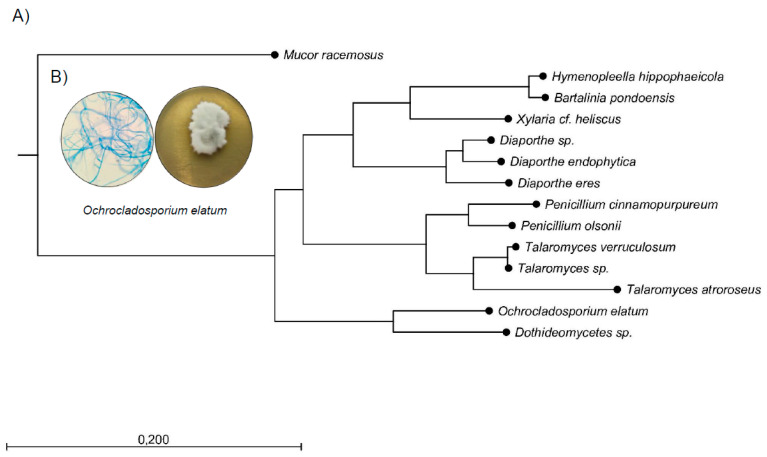
Phylogenetic analyses of endophytic fungi isolated from *S. terebinthifolius*. (**A**) Phylogenetic tree of maximum probability obtained from the analysis of restriction fragments of the ITS1 region. Numeric values indicate bootstrap support and later probabilities in this order. (**B**) Microscopy (100× magnification) and macromorphology of the endophytic fungus *O. elatum* in malt extract agar.

Sixteen endophytic fungal species were isolated from different parts of *S. terebinthifolius*, with 15 species identified (Table 1). Using morphological and sequencing approaches, five orders were found, as shown in Table 1: Eurotiales (6 species), Xylariales and Diaporthales (three species each), Pleosporales (two species) of the phylum Ascomycota, and Mucorales (one species) of the phylum Mucoromycota. The phylogenetic tree of the identified endophytic fungi is shown in Figure 1.

The frequency of colonization of the different parts of *S. terebenthifolius* is summarized in Table 2. Ten species showed a frequency of colonization above 5% and were selected for chemical and pharmacological studies. There was a distribution of colonization in the different parts evaluated, and only the fungus *B. pondoensis* was observed in all samples studied. The highest colonization frequencies were observed for *M. racemosus* in fruits, *Dothideomycetes* sp. in the stem, and *T. atroroseus* in roots.

### 3.2. Phenolic Compounds

The major phenolic compounds of the fungal extracts were hydroxybenzoic acids, followed by flavonols and hydroxycinnamic acids (Table 3). The extract of the endophytic fungus *O. elatum* (EFOe) showed the highest amounts of the phenolic compounds evaluated (Table 3).

### 3.3. Antioxidant Activity

The antioxidant properties of the endophytic fungi tested are summarized in Table 4. All extracts of the endophytic fungi from *S. terebinthifolius* exhibited antioxidant activity; however, the EFOe showed higher antioxidant activity than did the other extracts. The IC_50_ and EC_50_ values determined by the various methodologies used for EFOe are shown in Table 5.

### 3.4. Antibacterial Activity

The antibacterial activity of endophytic fungal extracts was tested against Gram-positive and Gram-negative bacteria of human pathogenic strains, namely, three ATCC strains and three methicillin-resistant nosocomial strains. All fungal extracts tested showed antibacterial activity against at least one human pathogenic bacterium (Table 6 and Table 7).

The fungal extracts showed selective activity against the growth of most bacterial strains (Table 6). Notably, for the Gram-positive *S. aureus*, ATCC and methicillin-resistant strains, only the extracts from the fungi *M. racemosus*, *D. endophytica*, *T. minioluteus*, *O. elatum* and *Dothideomycetes* sp. were able to inhibit their growth, with a minimum inhibitory concentration (MIC) between 0.5 and 1.0 mg/mL. In contrast, Gram-negative bacteria *K. pneumoniae* and *S. enteritidis*, ATCC strains, were sensitive to all fungal extracts evaluated, with MICs between 0.13 and 1.0 mg/mL. However, the *K. pneumoniae* and *S. enteritidis* nosocomial strains were sensitive only to the *D. endophytica* and *O. elatum* fungal extracts, with an MIC of 1.0 mg/mL. In summary, the *D. endophytica* and *O. elatum* extracts were active against the growth of all tested bacteria. The reference antibiotic gentamycin was not effective against the *S. aureus* ATCC and nosocomial strains and was active against the other *K. pneumoniae* and *S. enteritidis* strains.

The bactericidal activity of the fungal extracts, expressed by the minimum bactericidal concentration (MBC), is summarized in Table 7. Against *S. aureus*, ATCC and methicillin-resistant strains, only the *M. racemosus* and *O. elatum* strains had bactericidal activity, with MBCs between 0.5 and 1.0 mg/mL (Table 7). Bactericidal activity of all fungal extracts evaluated against ATCC strains of *K. pneumoniae* and *S. enteritidis* was observed, with MBCs between 0.25 and 1.0 mg/mL (Table 7). In contrast, none of the extracts exhibited bactericidal activity against the methicillin-resistant strains of these bacteria (Table 7).

### 3.5. Volatile Compounds

Volatile compounds were identified in the endophytic fungi extracts that showed the best biological activities in the antioxidant and antimicrobial assays. Among the identified volatile compounds, 1-dodecanol was the major compound in all extracts obtained, except in the fungal extract from *D. endophytica*, whose major compound was 2-phenylethanol (Table 8).

## 4. Discussion

During plant evolution, endophytic fungi contributed to the adaptive success of host plant species against abiotic and biotic environmental stressors. Endophytes are considered important sources of bioactive metabolites. They produce a variety of secondary metabolites, such as phenolic compounds, alkaloids, terpenoids, steroids and aromatic compounds, with various functions, such as antioxidant and defense activities (repellent or toxic) [35]. In this study, 15 endophytic fungi were identified in the fruits, stem bark and roots of *S. terebinthifolius*. For the first time for this species, fungi belonging to the genera *Mucor*, *Hymenopleella*, *Bartalinia*, *Talaromyces*, *Ochrocladosporium* and *Dothideomycetes* were identified. The identified endophytic fungi were able to produce phenolic compounds and volatile compounds with antioxidant and antimicrobial activities.

The distribution of the isolated fungi was not homogeneous in all investigated parts. The diversity of fungi present in the investigated parts, six to nine fungal species, was similar to that observed in *S. terebinthifolius* leaves by [28], who also found six endophytic fungal species, with the genera *Diaporthe*, *Penicillium*, and *Xylaria* being coincident between the leaves and the other parts investigated.

Some species were exclusive to different parts of the plant, such as *M. racemosus*, *P. olsonii* and *O. elatum* in the fruits, *H. hippohaeicol*, *X.* cf. *heliscus*, *D. eres* and *Dothideomycetes* sp. in stem bark and *P. cinnamopurpureum*, *Talaromyces* sp., *T. verruculosum* and *T. minioluteus* in the roots. Only the fungus *B. pondoensis* was present in the three investigated parts. The pleiotropic colonization of *B. pondoensis* may be linked to the action of this fungus on the endogenous levels of salicylic and jasmonic acids in the host plant [36], which are natural defense hormones against herbivory, a process that occurs in all parts of the plant.

The antioxidant activities of the fungal extracts observed may be related to the presence of the chemical compounds identified. Phenolic compounds have hydroxyl groups and have been described for their antioxidant potential due to their ability to eliminate free radicals, high reducing power and ability to inhibit lipid oxidation [37,38,39]. The phenolic compounds evaluated represent an important group of chemical substances. Hydroxybenzoic acids include gallic, p-hydroxybenzoic, protocatechuic, vanillic and syringic acids, whereas hydroxycinnamic acids are aromatic compounds whose most common representatives are caffeic, ferulic, p-coumaric and synaptic acids. Flavonols are a class of flavonoids whose main representatives are catechins, epicatechin, epicatechin-3-gallate, epigallocatechin and epigallocatechin-3-gallate. Data from the literature demonstrating the antioxidant capacity of fungal extracts from *Diaporthe* sp. [13] and *Dothideomycetes* sp. [40], extracted from other plant species, corroborate our results.

The highest concentrations of the chemical compounds evaluated were identified in the *O. elatum* fungal extract, which may be related to the best results that this fungus presented in the biological assays. Although the extracts from the fungi *O. elatum* and *T. verruculosum* showed similar concentrations of phenolic compounds, the antioxidant capacity of *O. elatum* was much higher in all tests performed. This difference in antioxidant capacity may be related to chemical diversity within the classes of chemical compounds identified. The highest antioxidant activity was observed in the fungus *O. elatum*, which allowed calculating the IC_50_ and EC_50_ for this extract only. Compared to the potency of the standard antioxidants ascorbic acid and BHA, the extract of the fungus *O. elatum* showed lower antioxidant activity, being approximately 10–30× less potent; however, notably, the antioxidants used as standards are isolated molecules, while the fungal extracts represent a set of substances at varied concentrations.

Endophytic fungi have also been identified as producers of antimicrobial substances, such as diaporthin and orthosporin isolated from the fungus *Diaporthe terebinthifolii*, with bacteriostatic action against Gram-positive and Gram-negative bacteria [41]. In our study, we observed that all isolated endophytic fungi showed bacteriostatic activity against Gram-positive ATCC strains of *K. pneumoniae* and *S. enteriditis*, whereas only *D. endophytica* and *O. elatum* were able to inhibit the growth of methicillin-resistant nosocomial strains. Regarding the Gram-negative bacteria *Staphylococcus aureus*, half of the evaluated fungi exhibited bacteriostatic activity against the two strains evaluated (ATCC and methicillin-resistant). In agreement with the bacteriostatic data, all fungal extracts were bactericidal against the ATCC strains of *K. pneumoniae* and *S. enteriditis*, but none demonstrated such an effect on methicillin-resistant nosocomial bacteria. These data are in agreement with previous studies in which the extracts from the endophytic fungi *Mucor racemosus* [42] and *Diaporthe* sp. [13] showed antibacterial activity against *K. pneumoniae*. However, data on the action of the other endophytic fungi isolated in this study against *S. enteritidis* bacteria are scarce in the literature.

The *M. rancemosus* and *O. elatum* fungi exhibited bactericidal activity against the *S. aureus* (Gram-negative) ATCC and methicillin-resistant nosocomial strains. In contrast, we did not observe antibacterial activity against *S. aureus*, as observed for the endophytic fungi *Diaporthe* sp. [13,43,44] and *Dothideomycetes* sp. [40] isolated from other plant species.

Antibacterial compounds can act on different targets in bacteria, including the cell wall, plasma membrane, protein synthesis, nucleic acid metabolism and DNA [45]. Among them, we highlight the phenolic compounds, which exhibit great structural diversity, such as the presence of hydroxyl groups as well as their substitution position and saturated side-chain length, which confers antibacterial activity to these compounds, allowing selective efficacy and effects against bacteria resistant to different antibiotics [46,47,48,49]. In a study of *S. terebinthifolius* leaves [27], the extracts isolated from the fungus *Alternaria* sp. showed the best antibacterial activity. This fungus belongs to the order Pleosporales, as does the fungus *O. elatum*, found in the fruits of this study and which also has the best antimicrobial activity.

Volatile compounds have also been described for their antimicrobial potential [50,51,52,53,54]. Among the endophytic fungi studied, *O. elatum* stood out for having the best antioxidant and antibacterial activities, probably attributed to phenolic compounds and volatile compounds. Among the volatile compounds identified in the fungal extract of *O. elatum*, 1-dodecanol was the major compound, followed by 3-methylbutyric acid, 2-phenylethanol, acetic acid and 2-phenylethyl acetate. The antibacterial activity of 1-dodecanol and 3-methylbutyric acid against resistant bacteria has been attributed to their aliphatic carbon chains, which act on the integrity of the plasma membrane [52,53,55]. The aromatic alcohol 2-phenylethanol [54], acetic acid [51] and 2-phenylethyl acetate [50] also have antibacterial activity against different Gram-positive and Gram-negative bacteria.

The high phenolic content of the fungal extract from *O. elatum* and of its volatile constituents, compared to the others, and its superior antioxidant and antimicrobial activities make it a promising source of molecules in future studies. However, the optimization of culture conditions such as pH, temperature and nitrogen and carbon sources, which improve the production of secondary metabolites [28], in future studies may increase the production capacity of secondary metabolites of *O. elatum* and increase the efficacy of its biological effects.

## 5. Conclusions

In conclusion, the present study expanded the knowledge on the diversity of endophytic fungal species known for *Schinus terebinthifolius*, describing species not yet identified, and showed, for the first time, the antioxidant and antimicrobial activities of several of these microorganisms, where the endophytic fungus *O. elatum* stood out. The antioxidant and antibacterial activities exhibited by the fungal extract from *O. elatum* can be attributed to the phenolic compounds and volatile compounds identified. Together, the results indicate that the fungal extract from *O. elatum* is a promising natural source of new antioxidant and antibacterial compounds.

## Figures and Tables

**Table 1:** Endophytic fungi isolated from *S. terebinthifolius* identified based on the BLASTed consensus sequence against the NCBI nucleotide database.

N°	Species	Order	NCBI	Identity (%)
1	*Mucor racemosus*	Mucorales	AJ271061.1	99
2	*Hymenopleella hippophaeicol*	Xylariales	KT949901.1	98
3	*Bartalinia pondoensis*	Xylariales	GU291796.1	96
4	*Xylaria* cf. *heliscus*	Xylariales	JQ760911.1	99
5	*Diaporthe* sp.	Diaporthales	KU747906.1	98
6	*Diaporthe endophytica*	Diaporthales	KX988294.1	99
7	*Diaporthe eres*	Diaporthales	HQ115664.1	96
8	*Penicillium cinnamopurpureum*	Eurotiales	AF033414.1	99
9	*Penicillium olsonii*	Eurotiales	EF200100.1	100
10	*Talaromyces* sp.	Eurotiales	JX244062.1	98
11	*Talaromyces verruculosum*	Eurotiales	AF510496.1	98
12	*Talaromyces atroroseus*	Eurotiales	LT558942.1	93
13	*Talaromyces minioluteus*	Eurotiales	AY213618.1	96
14	*Ochrocladosporium elatum*	Pleosporales	EU040233.1	96
15	*Dothideomycetes* sp.	Pleosporales	KX908640.1	97
16	Unknown	nd	nd	nd

nd: not detected.

**Table 2:** Frequency of colonization (%) and yield of the ethyl acetate extract of endophytic fungi isolated from fruits, stem bark and roots of *S. terebinthifolius*.

N°	Species	ESA Identification	Colonization Frequency (%)			Yield (mg)
			Fruits	Stem	Root	
1	*M.racemosus*	ESA-FE1	30.77	nd	nd	49.20
2	*H. hippophaeicol*	ESA-FE2	nd	2.53	nd	nd
3	*B. pondoensis*	ESA-FE3	15.38	8.86	6.82	13.00
4	*X.* cf. *Heliscus*	ESA-FE4	nd	1.27	nd	nd
5	*Diaporthe* sp.	ESA-FE5	15.38	27.85	nd	19.20
6	*D. endophytica*	ESA-FE6	15.38	10.13	nd	62.00
7	*D. eres*	ESA-FE7	nd	3.80	nd	nd
8	*P. cinnamopurpureum*	ESA-FE8	nd	nd	1.14	nd
9	*P. olsonii*	ESA-FE9	15.38	nd	nd	80.60
10	*Talaromyces* sp.	ESA-FE10	nd	nd	2.27	nd
11	*T. verruculosum*	ESA-FE11	nd	nd	18.18	182.00
12	*T. atroroseus*	ESA-FE12	nd	1.27	43.18	175.60
13	*T. minioluteus*	ESA-FE13	nd	nd	28.41	219.00
14	*O. elatum*	ESA-FE14	7.69	nd	nd	25.20
15	*Dothideomycetes* sp.	ESA-FE15	nd	43.04	nd	22.10
16	Unknown	-	nd	1.27	nd	nd

nd: not detected.

**Table 3:** Phenolic compounds of the extracts of the endophytic fungi isolated from *S. terebinthifolius* with a frequency of colonization above 5%.

Endophytic Fungi	Hydroxybenzoic Acids (mg GAE/g)	Hydroxycinnamic Acids (mg CAE/g)	Flavonols (mg QE/g)
*M. racemosus*	51.58 ± 1.33	6.99 ± 0.85	18.28 ± 1.02
*B. pondoensis*	70.48 ± 0.64	16.13 ± 0.83	46.85 ± 1.70
*Diaporthe* sp.	65.61 ± 0.74	14.74 ± 0.02	43.43 ± 0.93
*D.endophytica*	30.59 ± 1.55	0.95 ± 0.32	10.93 ± 0.46
*P. olsonii*	70.51 ± 0.80	10.44 ± 0.10	41.40 ± 0.69
*T. verruculosum*	75.52 ± 0.18	20.01 ± 0.17	76.51 ± 1.62
*T. atroroseus*	18.72 ± 0.74	0.00 ± 0.00	8.68 ± 0.56
*T. minioluteus*	21.66 ± 1.58	0.00 ± 0.00	5.08 ± 0.74
*O. elatum*	85.18 ± 2.73	25.18 ± 1.38	72.41 ± 4.35
*Dothideomycetes* sp.	58.72 ± 0.25	9.15 ± 0.08	28.62 ± 0.44

GAE: Gallic acid equivalent; CAE: caffeic acid equivalent; QE: quercetin equivalent. Results are expressed as mean ± SEM.

**Table 4:** Antioxidant properties of the extracts of the endophytic fungi from *S. terebinthifolius*.

Endophytic Fungi	DPPH (mg Trolox equivalent/g)	FRAP (mg Fe II equivalent/g)	β-carotene Bleaching Inhibition (%) (2 mg/mL)
*M. racemosus*	238.79 ± 18.43	166.11 ± 9.37	31.54 ± 0.88
*B. pondoensis*	80.35 ± 7.80	102.13 ± 1.67	29.36 ± 0.89
*Diaporthe* sp.	201.98 ± 16.37	153.40 ± 5.25	44.47 ± 0.20
*D. endophytica*	67.54 ± 2.23	46.30 ± 2.00	30.05 ± 0.66
*P. olsonii*	119.33 ± 7.24	82.18 ± 3.44	43.12 ± 0.09
*T. verruculosum*	206.16 ± 7.74	94.70 ± 1.90	28.69 ± 1.37
*T. atroroseus*	54.17 ± 7.46	30.62 ± 1.25	6.61 ± 0.29
*T. minioluteus*	91.32 ± 13.20	27.32 ± 0.52	4.65 ± 0.01
*O. elatum*	758.91 ± 0.01	272.72 ± 10.31	80.06 ± 0.01
*Dothideomycetes* sp.	151.08 ± 16.04	104.79 ± 11.85	37.86 ± 0.18

The results are expressed as mean ± SEM.

**Table 5:** Antioxidant properties of the extract of the endophytic fungus *O. elatum* (EFOe).

Sample	DPPH (IC_50_)	FRAP (EC_50_)	β-carotene Bleaching (IC_50_)
Ascorbic acid	7.41 ± 0.07	28.30 ± 0.27	nd
BHA	7.99 ± 0.14	55.78 ± 1.85	4.92 ± 0.21
EFOe	67.73 ± 0.16	539.14 ± 6.70	161.36 ± 19.07

IC_50:_ inhibitory concentration 50%; EC_50_: effective concentration 50%; nd: not detected. Results are expressed as mean ± SEM.

**Table 6:** Minimum inhibitory concentration (MIC) (mg/mL) of extracts from endophytic fungi isolated from *S. terebinthifolius*.

Endophytic Fungi	Gram-Positive Bacteria			Gram-Negative Bacteria			
	*Staphylococcus aureus*			*Klebsiella pneumoniae*		*Salmonella* *enteritidis*	
	ATCC 43300	ESA 66		ATCC 13883	ESA125	ATCC 13076	ESA 143
*M. racemosus*	1.00	1.00		0.25	nd	0.25	nd
*B. pondoensis*	nd	nd		0.25	nd	0.25	nd
*Diaporthe* sp.	nd	nd		0.50	nd	0.50	nd
*D. endophytica*	1.00	1.00		0.50	1.00	0.50	1.00
*P. olsonii*	nd	nd		0.25	nd	0.25	nd
*T. verruculosum*	nd	nd		0.25	nd	1.00	nd
*T. atroroseus*	nd	nd		0.50	nd	0.50	nd
*T. minioluteus*	1.00	1.00		0.50	nd	0.50	nd
*O. elatum*	0.50	0.50		0.13	1.00	0.50	1.00
*Dothideomycetes* sp.	1.00	1.00		0.25	nd	0.50	nd
Gentamicin	nd	nd		0.0002	0.0003	0.002	0.0002

ATCC: American Type Culture Collection; Hospital strain: ESA (Escola Superior Agrária of Bragança); nd, not detected (result higher 1.00 mg/mL).

**Table 7:** Minimum bactericidal concentration (MBC) (mg/mL) of the extracts from endophytic fungi isolated from *S. terebinthifolius*.

Endophytic Fungi	Gram-Positive Bacteria			Gram-Negative Bacteria			
	*Staphylococcus aureus*			*Klebsiella pneumoniae*		*Salmonella* *enteritidis*	
	ATCC 43300	ESA 66		ATCC 13883	ESA 125	ATCC 13076	ESA 143
*M. racemosus*	1.00	1.00		0.25	nd	0.25	nd
*B. pondoensis*	nd	nd		0.25	nd	0.50	nd
*Diaporthe* sp.	nd	nd		0.50	nd	0.50	nd
*D. endophytica*	nd	nd		0.50	nd	0.50	nd
*P. olsonii*	nd	nd		0.50	nd	0.25	nd
*T. verruculosum*	nd	nd		0.25	nd	1.00	nd
*T. atroroseus*	nd	nd		0.50	nd	0.50	nd
*T. minioluteus*	nd	nd		1.00	nd	0.50	nd
*O. elatum*	0.50	0.50		0.25	nd	0.50	nd
*Dothideomycetes* sp.	nd	nd		0.50	nd	0.50	nd
Gentamicin	nd	nd		0.0006	0.0003	0.002	0.002

ATCC: American Type Culture Collection; Hospital strain: ESA (Escola Superior Agrária of Bragança); nd, not detected (result higher 1.00 mg/mL).

**Table 8:** Volatile compounds of the endophytic fungi extracts from *S. terebinthifolius*.

(mg/L)	*RT*			Endophytic Fungi		
	(min)	*M. racemosus*	*D. endophytica*	*T. verruculosum*	*O. elatum*	*Dothideomycetes* sp.
Acetic acid	11.22	0.87	2.52	8.70	1.65	1.76
3-Methylbutyric acid	18.69	nd	nd	9.67	8.03	0.12
2-Phenylethyl acetate	24.05	1.66	1.87	0.26	0.18	0.10
2-Phenylethanol	27.02	4.17	56.01	1.55	1.97	0.46
1-Dodecanol	29.43	21.02	42.87	28.08	18.41	23.84
g-Decalactone	34.44	1.74	0.24	1.72	nd	nd
Hexadecanoic acid	54.77	nd	nd	13.96	nd	nd
4-Octanol (standard)	12.57	15.00	15.00	15.00	15.00	15.00

RT: retention time; nd: not detected.

## References

[1] Morton J.F. (1978). Brazilian pepper: Its impact on people, animals and the environment. Econ. Bot..

[2] Brandão M.G.L., Consenza G.P., Moreira R.A., Monte-Mor R.L.M. (2006). Medicinal plants and other botanical products from the Brazilian official pharmacopeia. Rev. Bras. Farmacogn..

[3] Tanvir R., Javeed A., Bajwa A.G. (2017). Endophyte bioprospecting in South Asian medicinal plants: An attractive resource for biopharmaceuticals. Appl. Microbiol. Biotechnol..

[4] Coulibaly A.Y., Hashim R., Sulaiman S.F., Sulaiman O., Ang L.Z., Ooi K.L. (2014). Bioprospecting medicinal plants for antioxidant components. Asian Pac. J. Trop. Med..

[5] Benko-Iseppon A.M., Crovella S. (2010). Ethnobotanical bioprospection of candidates for potential antimicrobial drugs from Brazilian plants: State of art and perspectives. Curr. Protein Pept. Sci..

[6] Aly A.H., Debbab A., Proksch P. (2011). Fungal endophytes: Unique plant inhabitants with great promises. Appl. Microbiol. Biotechnol..

[7] Strobel G., Daisy B., Castillo U., Harper J. (2004). Natural products from endophytic microorganisms. J. Nat. Prod..

[8] Kumar A., Patil D., Rajamohanan P.R., Ahmad A. (2013). Isolation, purification and characterization of vinblastine and vincristine from endophytic fungus *Fusarium oxysporum* isolated from *Catharanthus roseus*. PLoS ONE.

[9] Wu Y.Z., Zhang H.W., Sun Z.H., Daí J.G., Hu Y.C., Li R., Lin P.C., Xia G.Y., Wang L.Y., Qiu B.L. (2018). Bysspectin A, an unusual octaketide dimer and the precursor derivatives from the endophytic fungus *Byssochlamys spectabilis* IMM0002 and their biological activities. Eur. J. Med. Chem..

[10] Mani V.M., Soundari A.P., Karthiyaini D., Preeth K. (2015). Bioprospecting Endophytic Fungi and Their Metabolites from Medicinal Tree Aegle marmelos in Western Ghats, India. Mycobiology.

[11] Marseglia L., Manti S., D’Angelo G., Nicotera A., Parisi E., Di Rosa G., Gitto E., Arrigo T. (2014). Oxidative stress in obesity: A critical component in human diseases. Int. J. Mol. Sci..

[12] Zimta A.A., Cenariu D., Irimie A., Magdo L., Nabavi S.M., Atanasov A.G., Berindan-Neagoe I. (2019). The Role of Nrf2 Activity in Cancer Development and Progression. Cancers.

[13] Tanapichatsakul C., Monggoot S., Gentekaki E., Pripdeevech P. (2018). Antibacterial and Antioxidant Metabolites of *Diaporthe* spp. Isolated from Flowers of *Melodorum fruticosum*. Curr. Microbiol..

[14] Martin R.M., Bachman M.A. (2018). Colonization, Infection, and the Accessory Genome of *Klebsiella pneumoniae*. Front. Cell. Infect. Microbiol..

[15] Anderson C.J., Kendall M.M. (2017). *Salmonella enterica* serovar *Typhimurium* strategies for host adaptation. Front. Microbiol..

[16] Taylor T.A., Unakal C.G. (2017). Staphylococcus Aureus.

[17] Bhimji S.S., Unakal C.G. (2017). Bacteria, Gram Positive.

[18] Moellering R.C. (2012). MRSA: The first half century. J. Antimicrob. Chemother..

[19] Rocha P.S., Campos J.F., Souza V.N., Vieira M.C., Boleti A.P.A., Rabelo L.A., Santos E.L., Souza K.P. (2017). Antioxidant and Protective Effects of *Schinus terebinthifolius* Raddi Against Doxorubicin-Induced Toxicity. Appl. Biochem. Biotechnol..

[20] Silva M.M., Iriguchi E.K.K., Kassuya C.A.L., Vieira M.C., Foglio M.A., Carvalho J.E., Ruiz A.L.T.G., Souza K.P., Formagio A.S.N. (2017). *Schinus terebinthifolius*: Phenolic constituents and in vitro antioxidant, antiproliferative and in vivo anti-inflammatory activities. Rev. Bras. Farmacogn..

[21] D’Sousa C.O., Ribeiro P.R., Loureiro M.B., Simões R.C., de Castro R.D., Fernandez L.G. (2015). Phytochemical screening, antioxidant and antibacterial activities of extracts prepared from different tissues of *Schinus terebinthifolius* Raddi that occurs in the coast of Bahia, Brazil. Pharmacogn. Mag..

[22] Bendaoud H., Romdhane H., Souchard J.P., Cazaux S., Bouajila J. (2010). Chemical composition and anticancer and antioxidant activities of *Schinus molle* L. and *Schinus terebinthifolius* Raddi berries essential oils. J. Food Sci..

[23] El-Massry K.F., El-Ghorab A.H., Shaaban H.A., Shibamoto T. (2009). Chemical compositions and antioxidant/antimicrobial activities of various samples prepared from *Schinus terebinthifolius* leaves cultivated in Egypt. J. Agric. Food Chem..

[24] Silva G.B.P.G., Silvino K.F., Bezerra J.D.P., Farias T.G.S., Araújo J.M., Stamford T.L.M. (2017). Antimicrobial activity of *Phoma* sp. URM 7221: An endophyte from *Schinus terebinthifolius* Raddi (Anacardiaceae). Afr. J. Microbiol. Res..

[25] Cole E.R., Santos R.B., Lacerda Júnior V., Martins J.D., Greco S.J., Cunha Neto A. (2014). Chemical composition of essential oil from ripe fruit of *Schinus terebinthifolius* Raddi and evaluation of its activity against wild strains of hospital origin. Braz. J. Microbiol..

[26] Alves L.A., Freires I.A., Pereira T.M., Souza A., Lima E.O., Castro R.D. (2013). Effect of *Schinus terebinthifolius* on *Candida albicans* growth kinetics, cell wall formation and micromorphology. Acta Odontol. Scand..

[27] Tonial F., Maia B.H., Gomes-Figueiredo J.A., Sobottka A.M., Bertol C.D., Nepel A., Savi D.C., Vicente V.A., Gomes R.R., Glienke C. (2016). Influence of Culturing Conditions on Bioprospecting and the Antimicrobial Potential of Endophytic Fungi from *Schinus terebinthifolius*. Curr. Microbiol..

[28] Tonial F., Maia B.H., Sobottka A.M., Savi D.C., Vicente V.A., Gomes R.R., Glienke C. (2017). Biological activity of *Diaporthe terebinthifolii* extracts against *Phyllosticta citricarpa*. FEMS Microbiol. Lett..

[29] Kjer J., Debbab A., Aly A.H., Proksch P. (2010). Methods for isolation of marine-derived endophytic fungi and their bioactive secondary products. Nat. Protoc..

[30] Pinela J., Barros L., Carvalho A.M., Ferreira I.C. (2011). Influence of the drying method in the antioxidant potential and chemical composition of four shrubby flowering plants from the tribe Genisteae (Fabaceae). Food Chem. Toxicol..

[31] Bobo-García G., Davidov-Pardo G., Arroqui C., Vírseda P., Marín-Arroyo M.R., Navarro M. (2014). Intra-laboratory validation of microplate methods for total phenolic contente and antioxidant activity on polyphenolic extracts, and comparison with conventional spectrophotometric methods. J. Sci. Food Agric..

[32] Ustundag Y., Huysal K., Kahvecioglu S., Demirci H., Yavuz S., Sambel M., Unal D. (2016). Establishing reference values and evaluation of an in-house ferric reducing antioxidant power (FRAP) colorimetric assay in microplates. Eur. Res. J..

[33] Koleva I.I., Beek T.A., Linssen J.P.H., Groot A., Evstatieva L.N. (2002). Screening of Plant Extracts for Antioxidant Activity: A Comparative Study on Three Testing Methods. Phytochem. Anal..

[34] Molla Y., Nedi T., Tadesse G., Alemayehu H., Shibeshi W. (2016). Evaluation of the in vitro antibacterial activity of the solvent fractions of the leaves of *Rhamnus prinoides* L’Herit (Rhamnaceae) against pathogenic bactéria. BMC Complement. Altern. Med..

[35] Chandra S. (2012). Endophytic fungi: Novel sources of anticancer lead molecules. Appl. Microbiol. Biotechnol..

[36] Navarro-Meléndez A.L., Heil M. (2014). Symptomless endophytic fungi suppress endogenous levels of salicylic acid and interact with the jasmonate-dependent indirect defense traits of their host, lima bean (*Phaseolus lunatus*). J. Chem. Ecol..

[37] Amorati R., Valgimigli L. (2012). Modulation of the antioxidant activity of phenols by non-covalent interactions. Org. Biomol. Chem..

[38] Cheng Z., Li Y. (2004). Reducing power: The measure of antioxidant activities of reductant compounds?. Redox Rep..

[39] Rice-Evans C., Miller N., Paganga G. (1997). Antioxidant properties of phenolic compounds. Trends Plant. Sci..

[40] Leutou A.S., Yun K., Choi H.D., Kang J.S., Son B.W. (2012). New Production of 5-Bromotoluhydroquinone and 4-O-Methyltoluhydroquinone from the Marine-Derived Fungus *Dothideomycete* sp.. J. Microbiol. Biotechnol..

[41] De Medeiros A.G., Savi D.C., Mitra P., Shaaban K.A., Jha A.K., Thorson J.S., Rohr J., Glienke C. (2018). Bioprospecting of *Diaporthe terebinthifolii* LGMF907 for antimicrobial compounds. Folia Microbiol..

[42] Tajdini F., Amini M.A., Nafissi-Varcheh N., Faramarzi M.A. (2010). Production, physiochemical and antimicrobial properties of fungal chitosan from *Rhizomucor miehei* and *Mucor racemosus*. Int. J. Biol. Macromol..

[43] Sousa J.P.B., Aguilar-Perez M.M., Arnold A.E., Rios N., Coley P.D., Kursar T.A., Cubilla-Rios L. (2016). Chemical constituents and their antibacterial activity from the tropical endophytic fungus *Diaporthe* sp. F2934. J. Appl. Microbiol..

[44] Li G., Kusari S., Kusari P., Kayser O., Spiteller M. (2015). Endophytic *Diaporthe* sp. LG23 Produces a Potent Antibacterial Tetracyclic Triterpenoid. J. Nat. Prod..

[45] Calvo J., Martínez-Martínez L. (2009). Antimicrobial mechanisms of action. Enferm. Infecc. Microbiol. Clin..

[46] Martelli G., Giacomini D. (2018). Antibacterial and antioxidant activities for natural and synthetic dual-active compounds. Eur. J. Med. Chem..

[47] Rempe C.S., Burris K.P., Lenaghan S.C., Stewart C.N. (2017). The Potential of Systems Biology to Discover Antibacterial Mechanisms of Plant Phenolics. Front. Microbiol..

[48] Cushnie T.P., Lamb A.J. (2011). Recent advances in understanding the antibacterial properties of flavonoids. Int. J. Antimicrob. Agents.

[49] Cueva C., Moreno-Arribas M.V., Martín-Alvarez P.J., Bills G., Vicente M.F., Basilio A., Rivas C.L., Requena T., Rodríguez J.M., Bartolomé B. (2010). Antimicrobial activity of phenolic acids against commensal, probiotic and pathogenic bacteria. Res. Microbiol..

[50] Jirovetz L., Buchbauer G., Schmidt E., Denkova Z., Slavchev A., Stoyanova A., Geissler M. (2011). Purity, Antimicrobial Activities and Olfactory Evaluations of 2-Phenylethanol and Some Derivatives. J. Essent. Oil Res..

[51] Ryssel H., Kloeters O., Germann G., Schäfer T., Wiedemann G., Oehlbauer M. (2009). The antimicrobial effect of acetic acid-an alternative to common local antiseptics?. Burns.

[52] Hayashida-Soiza G., Uchida A., Mori N., Kuwahara Y., Ishida Y. (2008). Purification and characterization of antibacterial substances produced by a marine bacterium *Pseudoalteromonas haloplanktis* strain. J. Appl. Microbiol..

[53] Togashi N., Shiraishi A., Nishizaka M., Matsuoka K., Endo K., Hamashima H., Inoue Y. (2007). Antibacterial activity of long-chain fatty alcohols against *Staphylococcus aureus*. Molecules.

[54] Fraud S., Rees E.L., Mahenthiralingam E., Russell A.D., Maillard J.-Y. (2003). Aromatic alcohols and their effect on Gram-negative bacteria, cocci and mycobacteria. J. Antimicrob. Chemother..

[55] Yang X., Huang E., Yuan C., Zhang L., Yousef A.E. (2016). Isolation and Structural Elucidation of Brevibacillin, an Antimicrobial Lipopeptide from *Brevibacillus laterosporus* That Combats Drug-Resistant Gram-Positive Bacteria. Appl. Environ. Microbiol..

